# The Train Benchmark: cross-technology performance evaluation of continuous model queries

**DOI:** 10.1007/s10270-016-0571-8

**Published:** 2017-01-17

**Authors:** Gábor Szárnyas, Benedek Izsó, István Ráth, Dániel Varró

**Affiliations:** 1 Department of Measurement and Information Systems, Budapest University of Technology and Economics, Magyar tudósok krt. 2, Budapest, 1117 Hungary; 2 MTA-BME Lendület Research Group on Cyber-Physical Systems, Budapest, Hungary; 3 Department of Electrical and Computer Engineering, McGill University, Montreal, Canada; 4 IncQuery Labs Ltd., Bocskai út 77–79, Budapest, 1113 Hungary

**Keywords:** Well-formedness validation, Query evaluation, Performance benchmark, Graph databases, Semantic databases, Relational databases

## Abstract

In model-driven development of safety-critical systems (like automotive, avionics or railways), well-formedness of models is repeatedly validated in order to detect design flaws as early as possible. In many industrial tools, validation rules are still often implemented by a large amount of imperative model traversal code which makes those rule implementations complicated and hard to maintain. Additionally, as models are rapidly increasing in size and complexity, efficient execution of validation rules is challenging for the currently available tools. Checking well-formedness constraints can be captured by declarative queries over graph models, while model update operations can be specified as model transformations. This paper presents a benchmark for systematically assessing the scalability of validating and revalidating well-formedness constraints over large graph models. The benchmark defines well-formedness validation scenarios in the railway domain: a metamodel, an instance model generator and a set of well-formedness constraints captured by queries, fault injection and repair operations (imitating the work of systems engineers by model transformations). The benchmark focuses on the performance of query evaluation, i.e. its execution time and memory consumption, with a particular emphasis on reevaluation. We demonstrate that the benchmark can be adopted to various technologies and query engines, including modeling tools; relational, graph and semantic databases. The Train Benchmark is available as an open-source project with continuous builds from https://github.com/FTSRG/trainbenchmark.

## Introduction

Model-driven engineering of critical systems, like automotive, avionics or train control systems, necessitates the use of different kinds of models on multiple levels of abstraction and in various phases of development. Advanced design and verification tools aim to *simultaneously improve quality and decrease costs by early validation* to highlight conceptual design flaws well before traditional testing phases in accordance with the correct-by-construction principle. Furthermore, they improve productivity of engineers by automatically synthesizing different design artifacts (source code, configuration tables, test cases, fault trees, etc.) required by certification standards.

A challenging and critical subproblem in many design tools is the validation of well-formedness constraints and design rules of the domain. Industrial standard languages (e.g. UML, SysML) and platforms (e.g. AUTOSAR  [6], ARINC653 [2]) frequently define a large number of such constraints as part of the standard. For instance, the AADL standard [71] contains 75 constraints captured in the declarative Object Constraint Language (OCL) [54], while AUTOSAR defines more than 500 design rules.

As it is much more expensive to fix design flaws in the later stages of development, it is essential to detect violations of well-formedness constraints as soon as possible, i.e. immediately after the violation is introduced by an engineer or some automated model manipulation steps. Therefore, industrial design tools perform model validation by repeatedly checking constraints after certain model changes. Due to its analogy to *continuous integration* [17] used in source code repositories, we call this approach *continuous model validation*.

In practice, model validation is often addressed by using model query [86] or transformation engines: error cases are defined by model queries, the results of which can be automatically repaired by transformation steps. In practice, this is challenging due to two factors: (1) *instance model sizes* can grow very large as the complexity of systems under design is increasing [72], and (2) *validation constraints* get more and more sophisticated. As a consequence, validation of industrial models is challenging or may become infeasible.

To tackle increasingly large models, they are frequently split into multiple model fragments (as in open-source tools like ARTOP [5] or Papyrus [59]). This can be beneficial for *local constraints* which can be checked in the close context of a single model element. However, there are *global well-formedness constraints* in practice, which necessitate to traverse and investigate many model elements situated in multiple model fragments; thus, fragment-wise validation of models is insufficient.

As different underlying technologies are used in modeling tools for checking well-formedness constraints, assessing these technologies systematically on well-defined challenges and comparing their performance would be of high academic and industrial interest. In fact, similar scenarios occur when query techniques serve as a basis for calculating values of derived features [33], populating graphical views [15] or maintaining traceability links [33] frequently used in existing tools. Furthermore, runtime verification [45] of cyber-physical systems may also rely on incremental query systems or rule engines [31].

While there are a number of existing benchmarks for *query performance* over relational databases [16, 84] and triplestores [11, 30, 50, 73], workloads of modeling tools for validating well-formedness constraints are significantly different [40]. Specifically, modeling tools use *more complex queries* than typical transactional systems [43] and the perceived performance is more affected by *response time* (i.e. execution time for a specific operation such as validation or transformation) rather than throughput (i.e. the number of parallel transactions). Moreover, it is the worst-case performance of a query set which dominates practical usefulness rather than the average performance. Cases of *model transformation tool contests* [36, 48, 65, 69, 74, 88, 89] also qualify as set of benchmarks. However, their case studies do not consider the performance of incremental model revalidation after model changes.

In the paper, we define the Train Benchmark, a *cross-technology macrobenchmark* that aims to measure the performance of continuous model validation with graph-based models and constraints captured as queries.^1^ The Train Benchmark defines a scenario that is specifically modeled after *model validation* in modeling tools: at first, an automatically generated model (of increasing sizes) is loaded and validated; then, the model is changed by some transformations, which is immediately followed by the revalidation of constraints. The primary goal of the benchmark is to measure the execution time of each phase, while a secondary goal is a cross-technology assessment of existing modeling and query technologies that (could) drive the underlying implementation.

Railway applications often use MDE techniques [60] and rule-based validation [46]. This benchmark uses a domain-specific model of a railway system that originates from the MOGENTES EU FP7 [81] project, where both the metamodel and the well-formedness rules were defined by railway domain experts. However, we introduced additional well-formedness constraints which are structurally similar to constraints from the AUTOSAR domain [8].

The Train Benchmark intends to answer the following research questions:

1. How do existing query technologies scale for a continuous model validation scenario?
2. What technologies or approaches are efficient for continuous model validation?
3. What types of queries serve as performance bottleneck for different tools?

This paper systematically documents and extends previous versions of the Train Benchmark  [39] which were used in various papers [37, 38, 40, 78, 86]. A simplified version of the Train Benchmark (featuring only a single modeling language and scenario) was published in the 2015 Transformation Tool Contest [80]. The current paper documents the benchmark in depth and conceptually extends it by *several workload scenarios* assessed over *a set of queries and transformations* and adaptations to various graph-based models. Furthermore, the current paper provides a *cross-technology assessment* of *10 different open-source query tools* from *four substantially different modeling technologies*.

We designed the Train Benchmark to comply with the four criteria defined in [29] for domain-specific benchmarks.

1. *Relevance* It must measure the peak performance and price/performance of systems when performing typical operations within that problem domain.
2. *Portability* It should be easy to implement the benchmark on many different systems and architectures.
3. *Scalability* The benchmark should apply to small and large computer systems.
4. *Simplicity* The benchmark must be understandable, otherwise it lacks credibility.

The paper is structured as follows. Section 2 presents the metamodel and instance models used in the benchmark. Section 3 describes the workflow of the benchmark and specifies the scenarios, queries, transformations and the instance model generator. Section 4 shows the benchmark setup and discusses the results. Section 5 lists the related benchmarks from the semantic web, relational databases and MDE domains. Section 6 concludes the paper and outlines future research directions. Appendix 7 contains a detailed specification of the queries and transformations used in the benchmark.^2^

## Modeling and query technologies

Our cross-technology benchmark spans across four substantially different technological spaces, each with different metamodeling and model representation support. The tools also provide different query and transformation languages. This section introduces the domain model along the modeling and query technologies used in the benchmark.

### The domain model

**Fig. 1:**
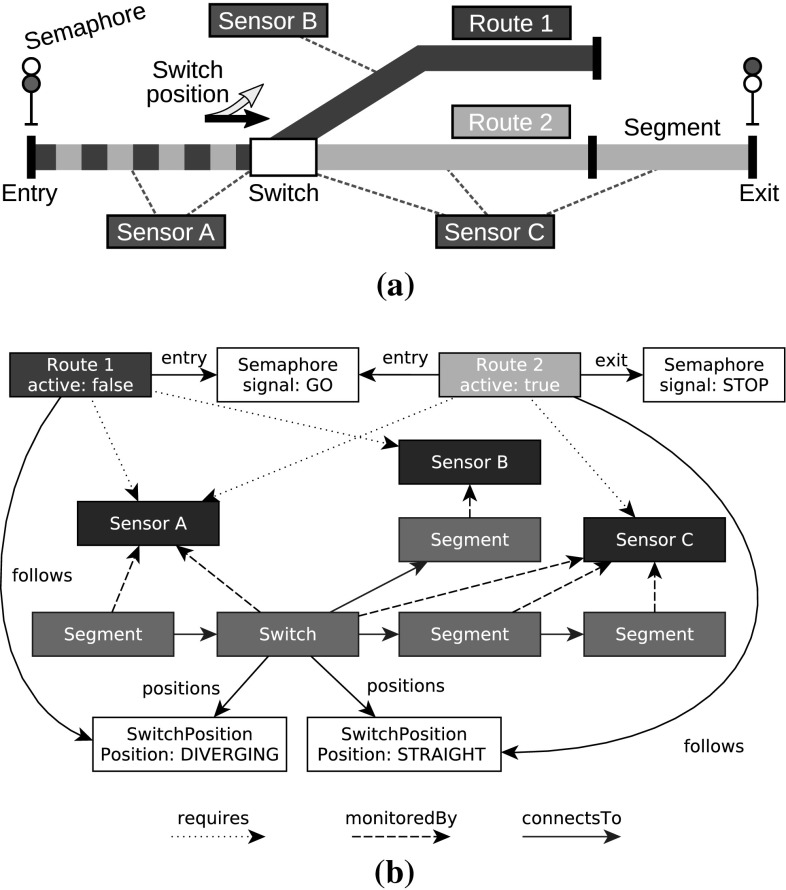
Domain concepts of the Train Benchmark. **a** Illustration for the concepts in the Train Benchmark models. **b** The concepts as a typed property graph

The goal of the Train Benchmark is to run performance measurements on a workload similar to validating a railway network model. Figure 1 shows a model of a simple railway network. Figure 1a illustrates the domain with a (partial) network, while Fig. 1b shows the same network as a graph.

In the context of the Train Benchmark, a train Route is a logical path of the railway, which requires a set of Sensors for safe operation. The occupancy of Track Elements (Segments and Switches) is monitored by sensors. A route follows certain Switch positions (straight or diverging) which describe the *prescribed* position of a switch belonging to the route. Different routes can specify different positions for the same switch. A route is active if all its switches are in the position prescribed by the switch positions followed by the route. Each route has a Semaphore on its entry and exit points.

### Modeling technologies

**Fig. 2:**
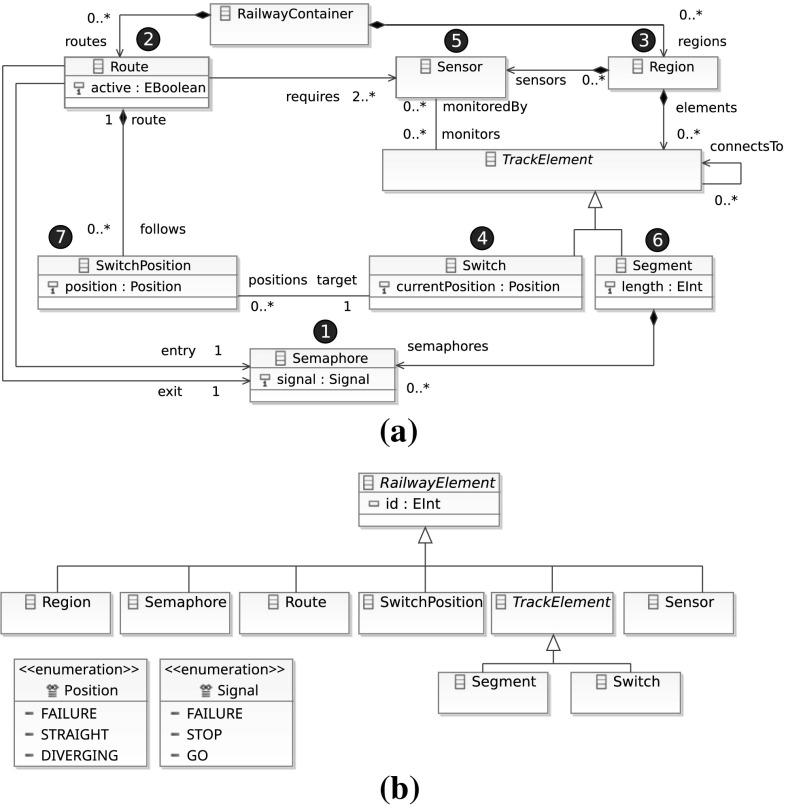
The metamodel of the Train Benchmark. **a** Containment hierarchy and references. **b** Supertype relations

Metamodeling is a technique for defining modeling languages where a metamodel specifies the abstract syntax (structure) of a modeling language. The metamodel of the Train Benchmark is shown in Fig. 2.

Table 1 defines the mapping from core object-oriented concepts to the various metamodeling frameworks used in the Train Benchmark. We discuss the following challenges for each modeling technology.

**Table 1:** Mapping object-oriented concepts to various representation technologies

OO	EMF	Property graphs	RDF	SQL
Class definition	EClass instance	Node label or type property	rdfs:Class	Table definition
Reference definition	EReference instance	Edge label	rdf:Property, owl:ObjectProperty	Foreign key definition
Attribute definition	EAttribute instance	Property name	rdf:Property, owl:DataTypeProperty	Column definition
Type	EDataType instance	(Only primitives)	rdfs:Datatype	(Only primitives)
Class attributes	eAttributes reference	$\Circle$	rdfs:domain	Table columns
Class references	eReferences reference	$\Circle$	rdfs:domain	Foreign keys
Attribute type	eAttributeType reference	property type	rdfs:range	Column type
Reference type	eReferenceType reference	$\Circle$	rdfs:range	$\Circle$
Superclasses	eSuperTypes reference	$\Circle$	rdfs:subClassOf	(Various mappings)
Aggregation	containment flag	$\Circle$	$\Circle$	$\Circle$

*Metamodeling* How does the technology define the metamodel, represent the classes and the supertype hierarchy?

*Instance models* How does the technology represent the instance models? For each technology, we present a simple instance model with a Segment (id: 1, length: 120), a Switch (id: 2, currentPosition: DIVERGING) and a connectsTo edge from the segment to the switch. Using this example, we also show how the unique identifiers are implemented across various domains. These identifiers are used for testing and ensuring deterministic results (cf. Sect. 3.8).

#### Eclipse Modeling Framework (EMF)

*Metamodeling* The Eclipse Modeling Framework provides Ecore, one of the *de facto* standard industrial metamodeling environments, used for defining several domain-specific languages and editors. Ecore enables to define metamodels and automates the generation of a wide range of tools. Ecore is discussed in detail in [18, 77].

**Fig. 3:**
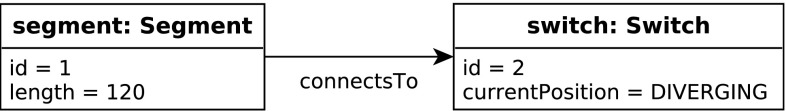
An EMF instance model

*Instance models* An EMF instance model is shown in Fig. 3. By default, EMF does not use numeric unique identifiers; instead (1) it uses references for the in-memory representation, and (2) it relies on XPath expressions for serialized models. However, developers may mark an attribute as an identifier. In the EMF metamodel of the Train Benchmark, we defined every class as a subtype of class RailwayElement which has an explicit id attribute, serving as a unique numeric identifier.

#### Property graphs

The property graph data model [68] extends typed graphs with properties (attributes) on the vertices and edges. This data model is common in NoSQL systems such as Neo4j [52], OrientDB [55] and Titan [83].

*Metamodeling* Graph databases provide no or weak metamodeling capabilities. Hence, models can either be stored in a weakly typed manner or the metamodel must be included in the graph (on the same metalevel as the instance model).

**Fig. 4:**
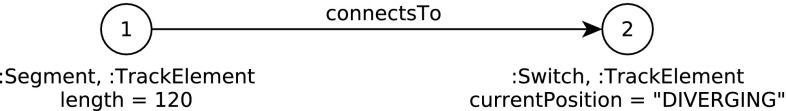
A property graph instance model

*Instance models* A property graph instance model is shown in Fig. 4. The vertices are typed with *labels*, e.g. vertex 1 is labeled as both Segment and TrackElement, while vertex 2 is labeled as both Switch and TrackElement.

The property graph data model requires nodes to have a unique (numeric) identifier. The ids are also persisted to the serialized model which makes them appropriate for testing the correctness of the queries.

#### Resource Description Framework (RDF)

The Resource Description Framework [96] is a family of W3C (World Wide Web Consortium) specifications originally designed as a *metadata data model*.

*Metamodeling* The RDF data model makes statements about *resources* (objects) in the form of triples. A *triple* is composed of a *subject*, a *predicate* and an *object*, e.g. “John is-type-of Person”, “John has-an-age-of 34”. Both the *ontology* (metamodel) and the *facts* (instance model) are represented as triples and stored together in the *knowledge base*.

The knowledge base is typically persisted in specialized databases tailored to store and process triples efficiently. Some triplestores are capable of *reasoning*, i.e. inferring logical consequences from a set of facts or axioms. RDF supports knowledge representation languages with different expressive power, ranging from RDFS [95] to OWL 2 [57].

**Fig. 5:**
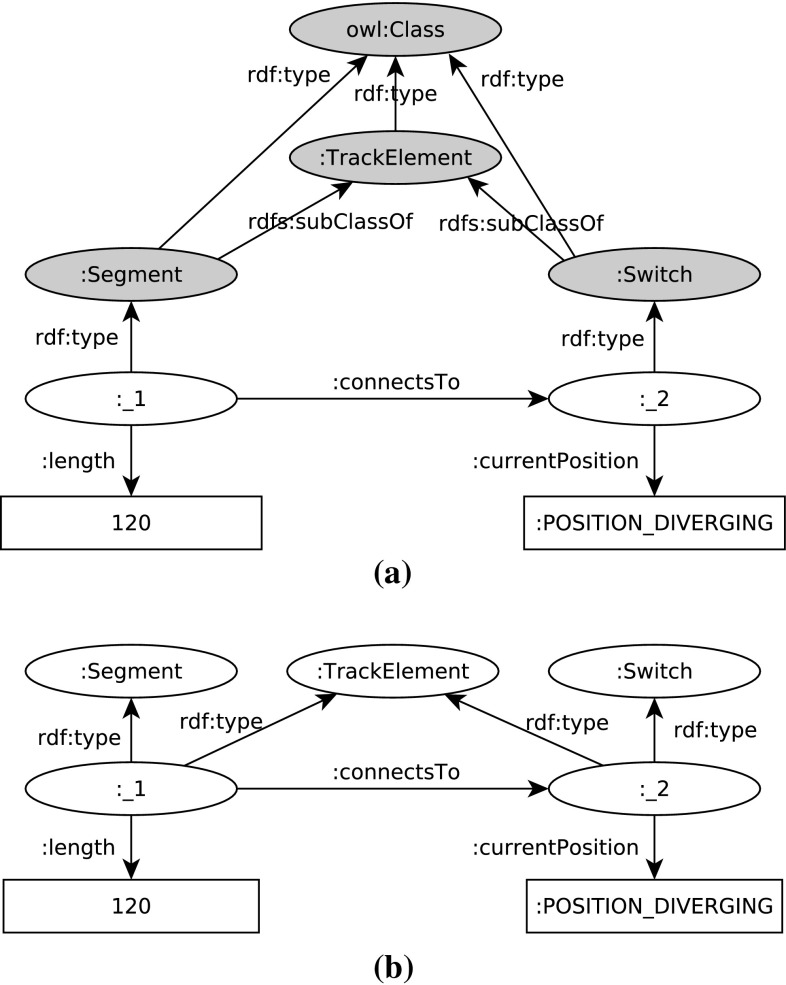
RDF instance models. **a** An RDF instance model *with metamodel*. The vertices for the (relevant part of the) metamodel are depicted in *gray*. **b** An RDF instance model *with inferred triples*. Note that the inferred edges to TrackElement node are explicitly asserted in the model

*Instance models* We provide two sets of RDF models:

- *RDF models with metamodel* The metamodel (described in OWL2 [57] and designed in Protégé [61]) is added to each instance model. Such an instance model is shown in Fig. 5a.
- *RDF models with inferred triples* For a resource of a given type, all supertypes are explicitly asserted in the model. For example, a resource with the type Segment also has the type TrackElement. Such an instance model is shown in Fig. 5b. Note that the _1 and _2 resources not only have the type Segment and Switch, but also the type TrackElement.

RDF uses Universal Resource Identifiers (URIs) to identify the resources. To assign a numeric identifier to each resource, the URIs follow the http://www.semanticweb.org/ontologies/2015/trainbenchmark#_x pattern, where x represents a unique identifier.

**Table 2:** Tools used in the benchmark

Format	Tool	Query language	Incremental	In-memory engine	Implementation language
EMF	Drools	DRL	$\CIRCLE$	$\CIRCLE$	Java
	Eclipse OCL	OCL	$\Circle$	$\CIRCLE$	Java
	EMF API	−	$\Circle$	$\CIRCLE$	Java
	Viatra Query	VQL	$\CIRCLE$	$\CIRCLE$	Java
graph	Neo4j	Cypher	$\Circle$	$\Circle$	Java
	TinkerGraph	−	$\Circle$	$\CIRCLE$	Java
RDF	Jena	SPARQL	$\Circle$	$\CIRCLE$	Java
	RDF4J	SPARQL	$\Circle$	$\CIRCLE$	Java
SQL	SQLite	SQL	$\Circle$	$\CIRCLE$	C
	MySQL	SQL	$\Circle$	$\Circle$	C++

#### Relational databases

Relational databases have been dominating the database landscape for the last 40 years with many free and commercial systems on the market. Due to the widespread adoption of the relational data model, these systems are mature and provide sophisticated tools for the administration tasks.

*Metamodeling* Object-to-relational mapping (ORM) is a well-known problem in software engineering [7]. The metamodel of the Train Benchmark is mapped to SQL tables with a standard ORM solution: each class is assigned to a separate table. A class and its superclass(es) are connected by using foreign keys. Many-to-many references are mapped to junction tables.

*Instance models* The instance models are stored as SQL dumps. The model uses primary keys for storing unique identifiers, defined as int attributes.

### Query technologies

We implemented the benchmark for a wide range of open-source tools operating on graph models with the exception of SQL (see Sect. 2.2 for the modeling technologies).

Table 2 shows the list of the implementations. We classify a tool *incremental* if it employs caching techniques and provides a dedicated incremental query evaluation algorithm that processes *changes* in the model and propagates these changes to query evaluation results in an incremental way (i.e. to avoid complete recalculations). Both Viatra Query and Drools are based on the Rete algorithm [41]. Eclipse OCL also has an incremental extension called the *OCL Impact Analyzer* [85]; however, it is not actively developed; therefore, it is excluded from the benchmark. In contrast, *non-incremental* tools use *search-based* algorithms. These algorithms evaluate queries with model traversal operations, which may be optimized using heuristics and/or caching mechanisms. The table also shows whether a tool uses an *in-memory engine* and lists the *implementation languages* of the tools.

#### EMF tools

- As a baseline, we have written a *local search-based* algorithm for each query in Java, using the EMF API. The implementations traverse the model without specific search plan optimizations, but they cut unnecessary search branches at the earliest possibility.
- The OCL [54] language is commonly used for querying EMF model instances in validation frameworks. It is a standardized navigation-based query language, applicable over a range of modeling formalisms. Taking advantage of the expressive features and widespread adoption of this query language, the project Eclipse OCL [19] provides a powerful query interface that evaluates such expressions over EMF models.
- Viatra Query  [8] is an Eclipse Modeling project where several authors of the current paper are involved. Viatra Query provides incremental query evaluation using the Rete algorithm [23]. Queries are defined in a graph pattern-based query language [10] and evaluated over EMF models. Viatra Query is developed with a focus on incremental query evaluation; however, it is also capable of evaluating queries with a local search-based algorithm [14].
- Incremental query evaluation is also supported by Drools [41], a rule engine developed by Red Hat. Similarly to Viatra Query, Drools is based on ReteOO, an object-oriented version of the Rete algorithm [23]. In particular, Drools 6 uses PHREAK, an improved version of ReteOO with support for lazy evaluation. Queries can be formalized using DRL, the Drools Rule Language. While Drools is not a dedicated EMF tool, the Drools implementation of the Train Benchmark works on EMF models. While EMF has some memory overhead [87], its advanced features, including deserialization and notifications, make it well suited for using with Drools.

#### RDF tools

Triplestores are usually queried via SPARQL (recursive acronym for SPARQL Protocol and RDF Query Language) [97] which is capable of defining graph patterns.

- Jena [3] is a Java framework for building Semantic Web and Linked Data applications. It provides an in-memory store and supports relational database backends.
- RDF4J [62] (formerly called Sesame) gives an API specification for many tools and also provides its own implementation.

**Fig. 6:**
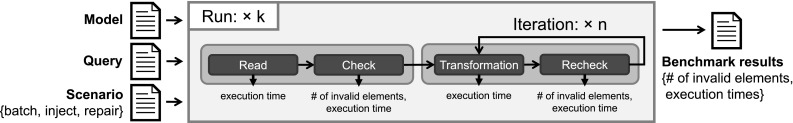
Phases of the benchmark

#### Property graph tools

We included two tools supporting the property graph data model:

- As of 2016, the most popular graph database is Neo4j [52] which provides multiple ways to query graphs: (1) a *low-level core API* for elementary graph operations, (2) the *Cypher language*, a declarative language focusing on graph pattern matching.
  While Cypher is very expressive and its optimization engine is being actively developed, it may be beneficial for some queries to implement the search algorithms manually [67, Chapter 6: Graph Database Internals].
- TinkerGraph is an in-memory reference implementation of the property graph interfaces provided by the Apache TinkerPop framework [4].

#### Relational databases

We included two popular relational database management systems (RDBMSs) to the benchmark.

- MySQL [51] is a well-known and widely used open-source RDBMS, implemented in C and C++.
- SQLite [56] is a popular embedded RDBMS, implemented in C.

## Benchmark specification

This section presents the specification of the Train Benchmark including inputs and outputs (Sect. 3.1), phases (Sect. 3.2), use case scenarios (Sect. 3.3), queries (Sect. 3.4), transformations (Sect. 3.5), a selected query with its transformations (Sect. 3.6), and instance models (Sect. 3.7).

### Inputs and outputs

*Inputs* A *benchmark case* configuration in the Train Benchmark takes a *scenario*, an *instance model size* and a *set of queries* as input. The specific *characteristics* of the model (e.g. error percentages) are determined by the scenario, while the *transformation* is defined based on the scenario and a query.

The *instance models* used in the Train Benchmark can be automatically generated using the generator module of the framework. The model generator uses a pseudorandom number generator with a fixed random seed to ensure the reproducibility of results (see Sect. 3.7 for details).

*Outputs* Upon the successful run of a benchmark case, the *execution times* of each phase and the *number of invalid elements* are recorded. Moreover, the collection of the element identifiers in the result set must be returned to allow the framework to check the correctness of the solution (Sect. 3.8). Furthermore, this result set also serves as a basis for executing transformations in the Repair scenario.

### Phases

In [8], we analyzed the performance of incremental graph query evaluation techniques. There, we defined four benchmark *phases* for model validation, depicted in Fig. 6.

1. During the read phase, the *instance model* is loaded from the disk to the memory and the *validation queries* are initialized (but not executed explicitly). The model has to be defined in one or more textual files (e.g. XMI, CSV, SQL dump), and binary formats are disallowed. The read phase includes the parsing of the input as well as the initialization of internal data structures of the tool.
2. In the check phase, the instance model is queried to identify invalid elements.
3. In the transformation phase, the model is changed to simulate the effects of model manipulations. The transformations are either performed on a subgraph specified by a simple pattern (Inject scenario) or on a subset of the model elements returned by the check phase (Repair scenario); see Sect. 3.3 for details.
4. The revalidation of the model is carried out in the recheck phase similarly to the check phase. The transformations modify the model to induce a change in the match set, which implies that the recheck phase will return a different match set than the previous check/recheck phases did.

### Use case scenarios

To increase the representativeness of the benchmark, we defined use case *scenarios* similar to typical workloads of real modeling tools, such as one-time validation (Batch scenario, used in [40, 87]), minor model changes introduced by an engineer (Inject scenario, used in [86]) or complex automated refactoring steps (Repair scenario, used in [78, 80]).

#### Batch validation scenario (Batch)

In this scenario, the instance model is loaded (read phase) from storage and a model validation is carried out by executing the queries in the check phase. This use case imitates a designer opening a model in an editor for the first time (e.g. after a checkout from a version control system) which includes an immediate validation of the model. In this scenario, the benchmark uses a model free of errors (i.e. no well-formedness constraints are violated), which is a common assumption for a model committed into a repository.

#### Fault injection scenario (Inject)

After an initial validation, this scenario repeatedly performs transformation and recheck phases. After the first validation (check), a small model manipulation step is performed (transformation), which is immediately followed by revalidation (recheck) to receive instantaneous feedback. The manipulation injects faults to the model; thus, the size of the match set *increases*.

Such scenario occurs in practice when engineers change the model in small increments using a domain-specific editor. These editors should detect design errors quickly and early in the development process to cut down verification costs according to the correct-by-construction principle.

#### Automated model repair scenario (Repair)

In this scenario, an initial validation is also followed by transformation and recheck phases. However, the model is repaired in the transformation phase based on the violations identified during the previous validation step. This is carried out by performing quick fix transformations [32]. Finally, the whole model is revalidated (recheck), and the remaining errors are reported. As the model manipulations fix errors in the model, the size of the match set *decreases*.

Efficient execution of this workload profile is necessary in practice for refactoring, incremental code generation, and model transformations within or between languages.

### Specification of queries

In the context of this paper, well-formedness constraints are captured and checked by *queries*. Each query identifies *violations of a specific constraint* in the model [8]. These constraints can be formulated in constraint languages (such as OCL), graph patterns and as relational queries.

In the check and recheck phases of the benchmark, we perform a *query* to retrieve the elements violating the well-formedness constraint defined by the benchmark case. The complexity of queries ranges from simple property checks to complex path constraints consisting of several navigation operations. The graph patterns are defined with the following syntax and semantics.

- *Positive conditions* define the structure and type of the vertices and edges that must be satisfied.
- *Negative conditions* (also known as negative application conditions) define subpatterns which must not be satisfied. Negative conditions are displayed in a red rectangle with the NEG caption.
- *Filter conditions* are defined to check the value of vertex properties. Filter conditions are typeset in *italic*.

**Fig. 7:**
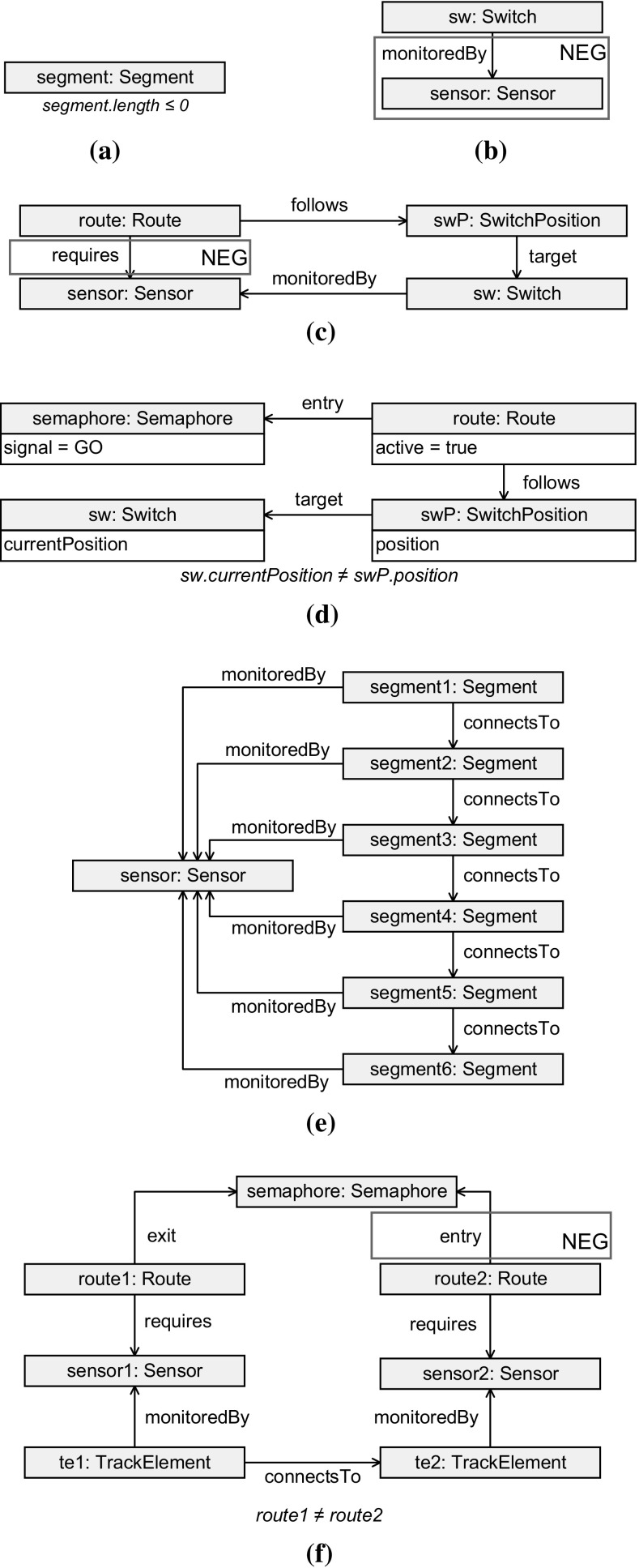
The patterns of benchmark queries. **a** The PosLength pattern. **b** The SwitchMonitored pattern. **c** The RouteSensor pattern. **d** The SwitchSet pattern. **e** The ConnectedSegments pattern. **f** The SemaphoreNeighbor pattern

We define the following six constraints by graph patterns (see Fig. 7). Each corresponding query checks a specific constraint and covers some typical query language features.

- PosLength (Fig. 7a) requires that a segment must have a positive length. The corresponding query defines a simple property check, a common use case in validation.
- SwitchMonitored (Fig. 7b) requires every switch to have at least one sensor connected to it. The corresponding query checks whether a vertex is connected to another vertex. This pattern is common in more complex queries, e.g. it is used in the RouteSensor and SemaphoreNeighbor queries.
- RouteSensor (Fig. 7c) requires that all sensors associated with a switch that belongs to a route must also be associated directly with the same route. The corresponding query checks for the absence of circles, so the efficiency of performing navigation and evaluating negative conditions is tested.
- SwitchSet (Fig. 7d) requires that an entry semaphore of an active route may show GO only if all switches along the route are in the position prescribed by the route. The corresponding query tests the efficiency of navigation and filtering operations.
- ConnectedSegments (Fig. 7e) requires each sensor to have at most 5 segments. The corresponding query checks for “chains” similar to a transitive closure. This is a common use case in model validation.
- SemaphoreNeighbor (Fig. 7f) requires routes which are connected through a pair of sensors and a pair of track elements to belong to the same semaphore. The corresponding query checks for the absence of circles, so the efficiency of join and antijoin [76] operations is tested. One-way navigable references are also present in the constraint, so the efficiency of their evaluation is also measured. Subsumption inference is required, as the two track elements (te1, te2) can be switches or segments.

*Structural similarity to AUTOSAR.* Several of these queries are adapted from constraints of the AUTOSAR standard [6] and represent common validation tasks such as attribute and reference checks or cycle detection. In accordance with our previous paper [8], the following graph patterns are strongly inspired by AUTOSAR constraints, i.e. the matching subgraphs of corresponding graph queries are either isomorphic or structurally similar:

- RouteSensor $\leftrightarrow$ simple physical channel: both check for the absence of a circle through edges with many-to-one and many-to-many cardinalities.
- SemaphoreNeighbor $\leftrightarrow$ signal group mapping: both check for the absence of a circle of 7 elements.
- SwitchMonitored $\leftrightarrow$ ISignal: both check a negative application condition for a single element.

**Table 3:** Description of the metrics in the benchmark

	PosLength	SwitchMonitored	RouteSensor	SwitchSet	ConnectedSegments	SemaphoreNeighbor
# parameters	2	1	4	6	7	7
# variables	2	2	4	6	7	7
# vertex types	1	2	4	4	2	4
# attributes	1	0	0	4	0	0
# attribute and equality checks	1	0	0	3	0	1
# edge constraints	0	0	3	3	11	6
# negative conditions	0	1	1	0	0	1

The query metrics adapted from [40, 87] are listed in Table 3. The metrics indicate that all relevant features of query languages are covered by our queries except for transitive closure and recursion. We decided to omit these features from the benchmark as they are supported by only a few query technologies.

### Specification of transformations

To capture complex operations in the scenarios, we use graph transformation rules [70] which consist of (1) a precondition pattern captured as a graph query and (2) an action with a sequence of elementary graph manipulation operations. The transformations are defined with a syntax similar to tools such as GROOVE, FUJABA [53] and Viatra2  [94]. For defining the patterns and transformations, we used a graphical syntax similar to GROOVE [64]:

- *Inserting* new vertices and new edges between existing vertices (marked with «new»).
- *Deleting* existing vertices and edges (marked with «del»). The deletion of a vertex implies the deletion of all of its edges to eliminate dangling edges.
- *Updating* the properties of a vertex (noted as *property* $\leftarrow$ *new value*).

Our transformations cover all elementary model manipulation operations, including the insertion and deletion of vertices and edges, as well as the update of attributes. A detailed specification of the queries and transformation is given in Appendix 7. In this section, we only discuss the RouteSensor query and its transformations in detail.

### Query and transformations for constraint RouteSensor

We present the specification of query RouteSensor and its related transformations used in the benchmark.

*Description* To check whether constraint RouteSensor (see Sect. 3.4) is violated, the query (Fig. 7c) looks for routes (route) that follow a switch position (swP) connected to a sensor (sensor) via a switch (sw), but without a requires edge from the route to the sensor.

*Inject transformation* Random requires edges are removed.

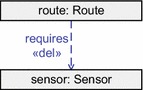



*Repair transformation* The missing requires that edge is inserted from the route to the sensor in the match, which fixes the violation of the constraint.

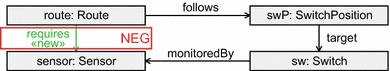



### Instance model generation and fault injection

To assess scalability, the benchmark uses instance models of growing sizes where each model contains twice as many model elements as the previous one. The sizes of instance models follow powers of two (1, 2, 4, ..., 2048): the smallest model contains about 5000 triples, and the largest one (in this paper) contains about 19 million triples.

The instance models are systematically generated based on the metamodel: first, small instance model fragments are generated; then, they are connected to each other. To avoid highly symmetric models, the exact number of elements and cardinalities is randomized to make it difficult for query tools to efficiently cache models.

The instance model generator is implemented in an imperative manner. The model is generated with nested loops, where each loop generates a specific element in an order driven by the containment hierarchy. In Fig. 2a, we annotated the metamodel to include the order of generating elements: (1) *semaphores*, (2) *routes*, (3) *regions*, (4) *switches*, (5) *sensors*, (6) *segments* and (7) *switch positions*.

The fault injection algorithm works as follows. For each well-formedness constraint, we select a model element which could introduce a violation of that constraint. For example, compared to a well-formed model, the violations are injected as follows.

- Constraint PosLength is violated by assigning an invalid value to the length attribute.
- Constraint SwitchMonitored is violated by deleting all monitoredBy edges of a Switch.
- Constraint RouteSensor is violated by deleting the requires edge from a Route to a Sensor.
- Constraint SwitchSet is violated by setting an invalid currentPosition attribute to a Switch (i.e. not the position of the corresponding SwitchPosition followed by the Route).
- Constraint SemaphoreNeighbor is violated by deleting an entry edge between a Route and a Semaphore.
- Constraint ConnectedSegments is violated by adding an additional (sixth) Segment to the same Sensor and connecting it to the last Segment.

The generator injects these faults with a certain probability (Table 4) using a random generator with a predefined random seed. These errors are found and reported in the check phase of the benchmark.

**Table 4:** Error probabilities in the generated instance model

Constraint	Batch (%)	Inject (%)	Repair (%)
PosLength	0	2	10
SwitchMonitored	0	2	18
RouteSensor	0	4	10
SwitchSet	0	8	15
ConnectedSegments	0	5	5
SemaphoreNeighbor	0	7	25

### Ensuring deterministic results

During transformation phase of the Repair scenario, some invalid submodels (i.e. pattern matches) are selected and repaired. In order to ensure *deterministic, repeatable results*:

- The elements for transformation are chosen using a pseudorandom generator with a fixed random seed.
- The elements are always selected from a deterministically *sorted list*.

The matches may be returned in any collection in any order, given that the collection is unique. The matches are interpreted as *tuples*, e.g. the RouteSensor query returns $\mathsf \langle route, sensor, swP, sw \rangle$ tuples. The tuples are sorted using a lexicographical ordering.

The ordered list is used to ensure that the transformations are performed on the same model elements, regardless of the return order of the match set. Neither ordering nor sorting is included in the execution time measurements.

## Evaluation

In this section, we discuss the benchmark methodology, present the benchmark environment and analyze the results. For implementation details, source code and raw results, see the benchmark website.^3^

### Benchmark parameters

A *measurement* is defined by a certain *tool* (with its *parameters*), *scenario*, *model size*, *queries* and *transformations*.

Table 5 shows the tools, parameters, scenarios, queries and sizes used in the benchmark. If a tool has no parameters, it is only executed once, otherwise it is executed with each optional parameter.

**Table 5:** Configuration parameters

Parameter	Values	Details in
(a) Benchmark-specific parameters		
Scenario	Batch	Section 3.3.1
	Inject	Section 3.3.2
	Repair	Section 3.3.3
Queries	ConnectedSegments	Section 7.1
	PosLength	Section 7.2
	RouteSensor	Section 3.6
	SemaphoreNeighbor	Section 7.4
	SwitchMonitored	Section 7.5
	SwitchSet	Section 7.6
Size	$1, 2, 4, \ldots , 2048$	Section 3.7

Tool	Version	Parameters
(b) Tool-specific parameters		
Drools	6.5.0	–
Eclipse OCL	3.3.0	–
EMF API	2.10.0	–
Jena	3.0.0	No inferencing
		inferencing
MySQL	5.7.16	–
Neo4j	3.0.4	Core API
		Cypher
RDF4J	2.1	No inferencing
SQLite	3.8.11.2	–
TinkerGraph	3.2.3	–
Viatra Query	1.4.0	Local search
		Incremental

**Fig. 8:**
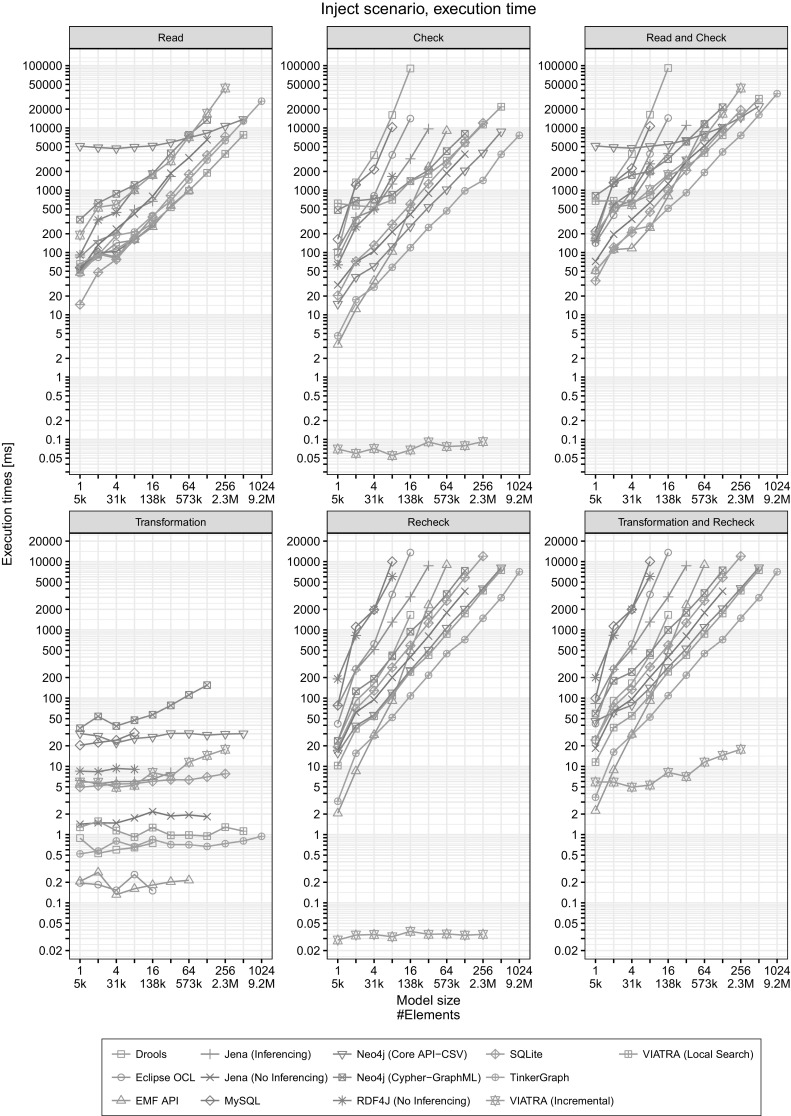
Execution times in the Inject scenario

**Fig. 9:**
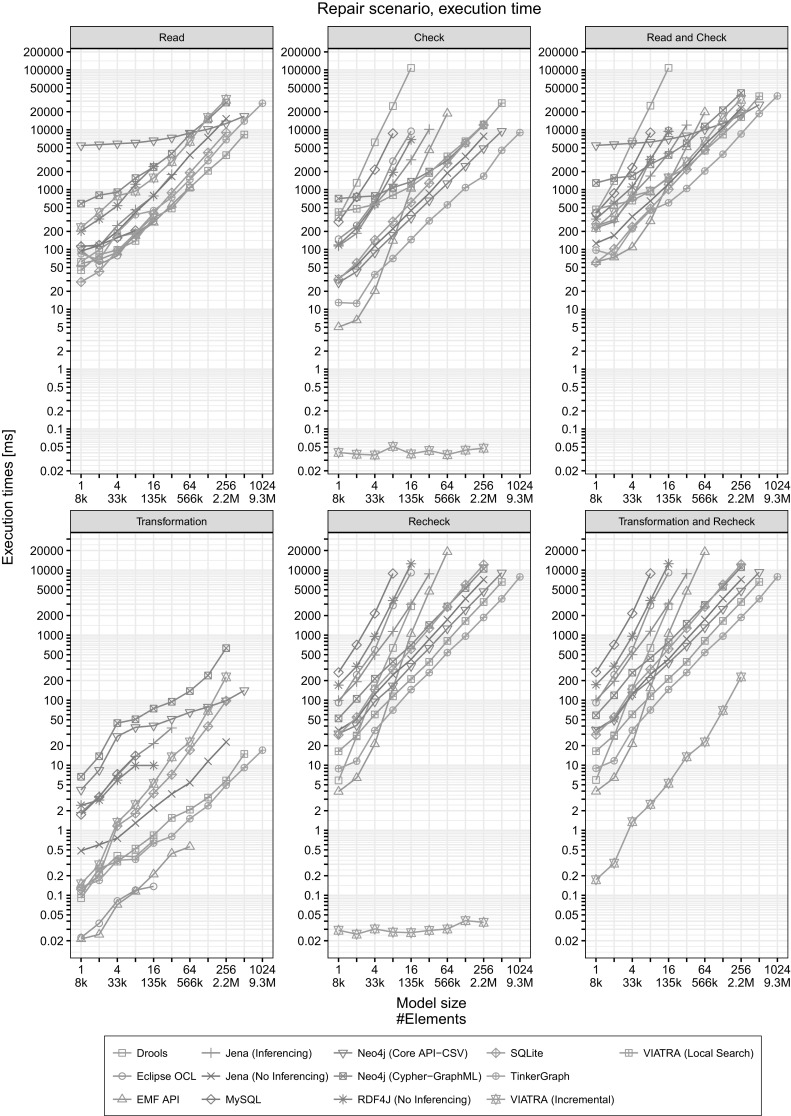
Execution times in the Repair scenario

### Execution of benchmark runs and environment

#### Benchmark scenarios

We investigated the following benchmark scenarios:

Batch *scenario* We executed the Batch scenario with all six queries (Sect. 3.4) to approximate memory consumption. The results are shown in Fig. 11.

Inject *scenario*

1. The benchmark loads the model and evaluates the *queries* as *initial validation*, and we measure execution times for read, check and their sum. The results are shown in the left column of Fig. 8.
2. The benchmark iteratively performs the Inject transformations for each query 10 times (Fig. 6, $n = 10$) followed by an immediate recheck step in each iteration. The transformation modifies a *fixed* number of elements (10) in each iteration. We measure the mean execution time for continuous validation for each phase (transformation, recheck and their sum). The results are shown in the right column of Fig. 8.

Repair *scenario.*

1. The benchmark performs the initial validation similarly to the Inject phase. The execution times for read, check and their sum are listed in the left column of Fig. 9.
2. The benchmark iteratively performs the Repair transformation for each query 8 times (Fig. 6, $n = 8$) followed by an immediate recheck step in each iteration. The transformation modifies a *proportional* amount of the invalid elements (5%). We measure the mean execution time for continuous validation for each phase (transformation, recheck and their sum). The results shown in the right column of Fig. 9.

#### Benchmark environment

The benchmark was performed on a virtual machine with an eight-core, 2.4 GHz Intel Xeon E5-2630L CPU with 16 GB of RAM, and an SSD hard drive. The machine was running a 64-bit Ubuntu 14.04 server operating system and the Oracle JDK 1.8.0_111 runtime. The independence of performance measurements was guaranteed by running each sequentially and in a separate JVM.

### Measurement of execution times

If all runs are completed within a *timeout* of 15 minutes, the measurement is considered successful and the *measurement results* are saved. If the measurement does not finish within the time limit, its process is terminated and its results are discarded. The results were processed as follows.

1. The *mean* execution time was calculated for each phase. For example, in the Repair scenario, the execution times of the transformation and the recheck phases are determined by their *average* execution time. This is determined independently for all runs.
2. For each phase, the *median* value of the 5 runs was taken.

Using the mean value to the describe the execution time of repeated transformation and recheck phases is aligned with the recommendations of [22]. Moreover, from a statistical perspective, taking the median value of the sequential runs can better compensate for transients potentially perceived during a measurement.

For measuring the execution times, the heap memory limit for the Java Virtual Machine (JVM) was set to 12 GB.

#### How to read the charts?

*Detailed plots* The plots in Figs. 8 and 9 present the execution times of a certain workload with respect to the model size. Each plot can be directly interpreted as *an overall evaluation of execution time against increasing model sizes* dominated by the worst-case behavior of a tool.

On each plot, the horizontal axis (with base 2 logarithmic scale) shows the model size and the vertical axis (with base 10 logarithmic scale) shows the execution time of a certain operation. Note that as the execution time of phases varies greatly (e.g. the read phase takes longer than the check phase as it contains disk operations), the vertical axes on the plots *do not use the same scale*, i.e. the minimum and maximum values are adjusted to make the plots easier to read.

The logarithmic scales imply that a “linear” appearance of all measurement series correspond to a (low-order) polynomial *O* characteristic where the slope of a plot determines the dominant order (exponent). Moreover, a constant difference on a plot corresponds to a constant order-of-magnitude difference. However, different plots are not directly comparable to each other visually due to the different scales.

**Fig. 10:**
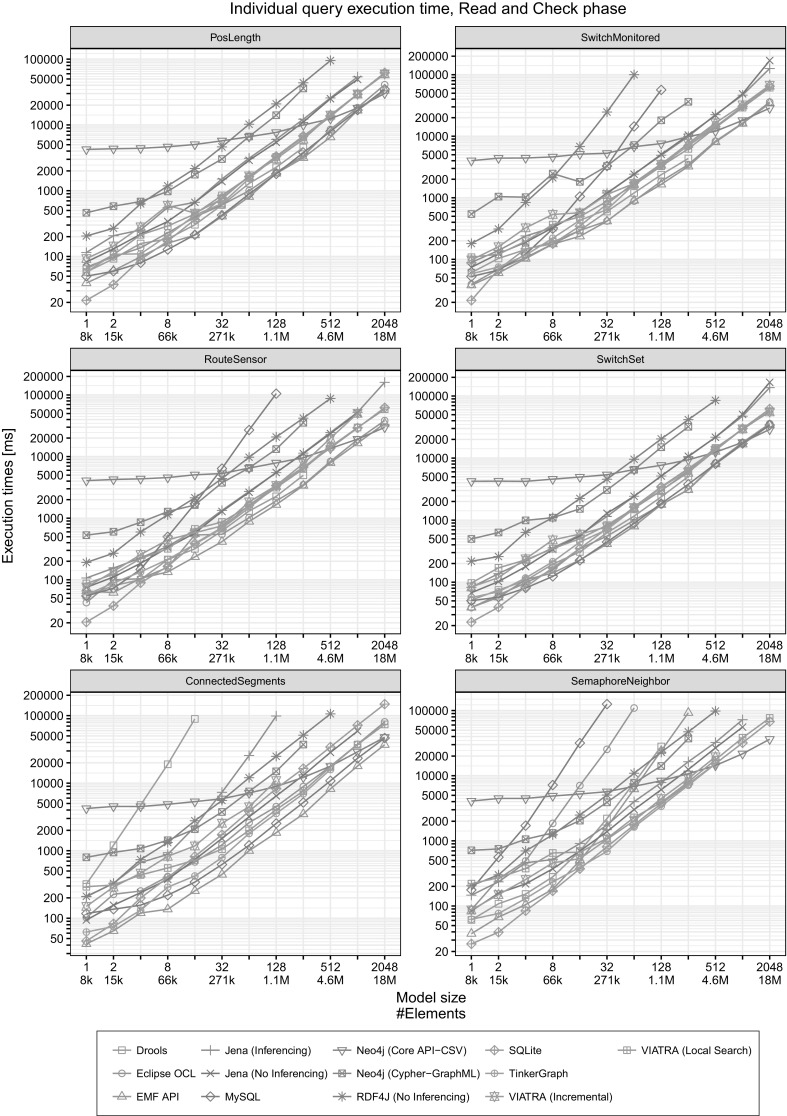
Execution times for individual queries (Read and Check)

*Individual query plots* The plots in Fig. 10 help us identify *specific strength and weaknesses of different tools* and highlight which query turned out to be the performance bottleneck. This can explain why certain tools had a timeout even for medium-sized models in the detailed plots of Figs. 8 and 9.

### Measurement of memory consumption

Determining the memory consumption of applications running in managed environments (such as the JVM) is a challenging task due to (1) the non-deterministic nature of the garbage collector and (2) sophisticated optimizations in collection frameworks which often allocate memory in advance and only free memory when it is necessary [12].

**Fig. 11:**
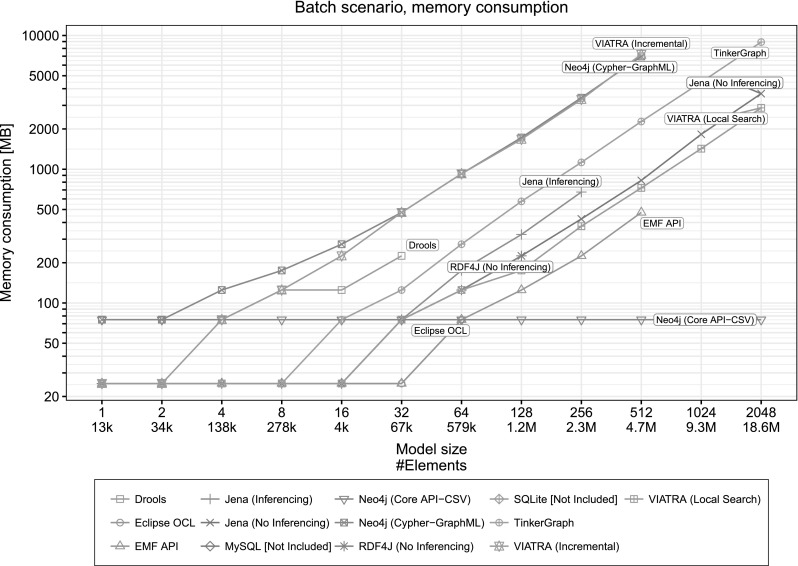
Estimated memory consumption for loading the model and evaluating all queries. (Memory consumption in this figure at a certain model size does not correspond to the execution times of Figs. 8–10.)

For a reliable estimation on memory consumption, we used the following approach.

1. We set a hard limit *L* to the available heap memory for the JVM and perform a *trial* of the run.
2. Based on the result of the trial, we either decrease or increase the limit.
  - If the trial successfully executed within the specified timeout, we decrease the limit to $L' = L/2$.
  - If the execution failed (due to memory exhaustion or timeout), we increase the limit to $L' = 2L$.

This results in a binary search-like algorithm, which ensures a resolution of $L_{\mathrm {initial}} / 2^{t-1}$, given an initial limit $L_{\mathrm {initial}}$ and *t* trials. For example, with an initial limit of 6.4 GB of memory and 9 trials, this approach provides a resolution of $6400~\mathrm {MB}/2^8 = 25~\mathrm {MB}$ (as used in our measurements later).

The results are shown in Fig. 11.^4^ Note that the measurements for execution time and memory consumptions were performed separately. The measurements in Figs. 8, 9, and 10 used a larger, fixed amount of memory. For instance, the low memory consumption of Neo4j in Fig. 11 corresponds to significantly larger execution time than reported in Fig. 10.

The results show that incremental tools (in particular, Viatra Query, in incremental mode) use more memory than non-incremental ones. This is expected as incremental tools utilize space–time tradeoff, i.e. they trade memory for execution speed by building caches of the interim query results and use it for efficient recalculations.

*Summary figures* We provide heatmaps to summarize results on execution times. We divided the model sizes to three categories: small (less than 100k triples), medium (100k–1M triples) and large (more than 1M triples), while the execution times were partitioned to instantaneous (less than 0.2 s), fast (0.2...1 s), acceptable (1...5 s) or slow (more than 5 s). The cells of the heatmaps represent the relative frequency of a particular model size–execution time combination with a darker color indicating more tools belonging to that combination. For example, a darker lower left cell (small/instantaneous) indicates that most tools perform the operation almost instantly for small models.

**Fig. 12:**
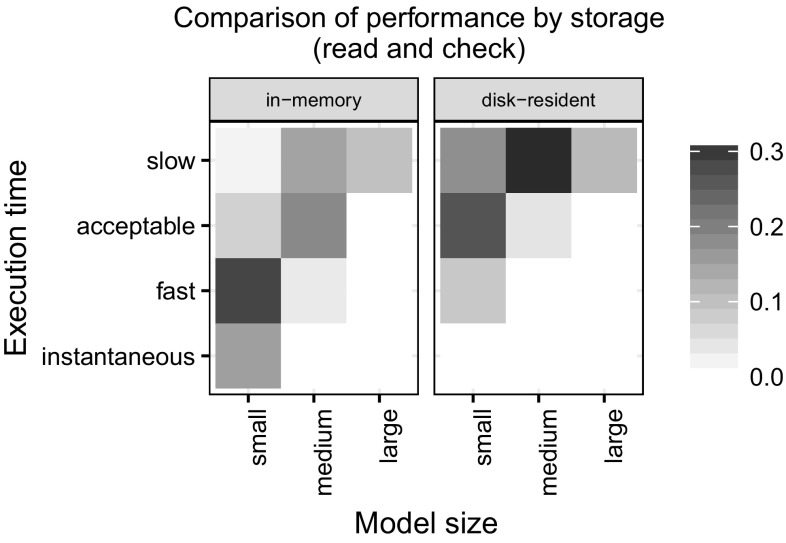
Comparison of performance by storage

**Fig. 13:**
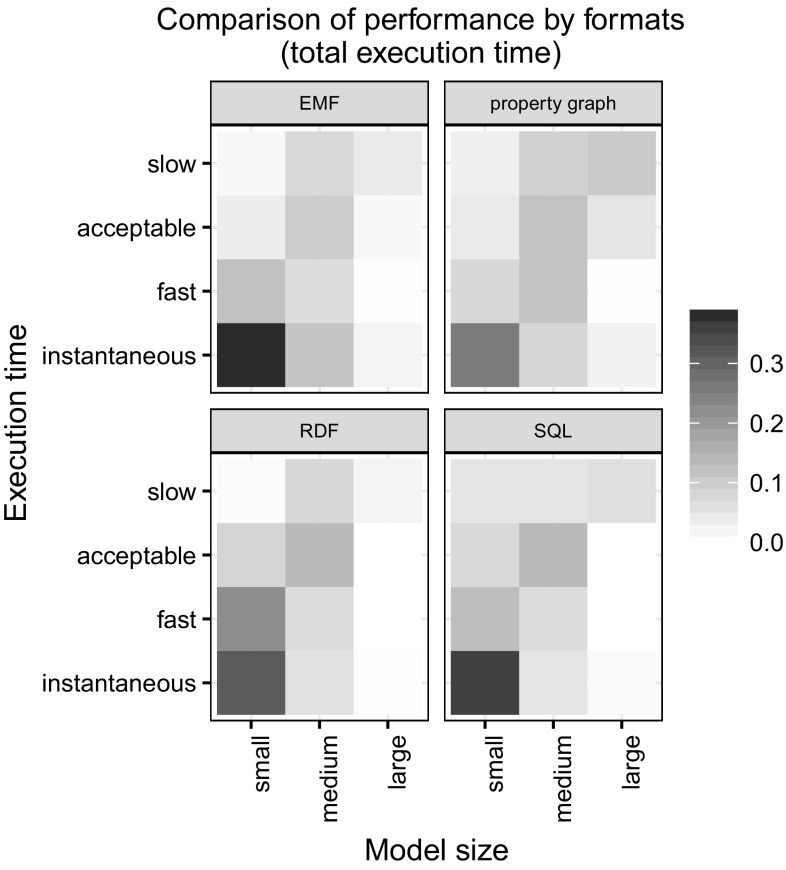
Comparison of performance by formats

- Figure 12 compares disk-based and in-memory databases. As expected, in-memory databases provide better response times in general.
- Figure 13 shows the formats. It shows that tools using EMF implementations perform very well on small models and also scale for large models. SQL and property graph implementations show moderate performance. RDF implementations are slower for small models and do not scale for large models.

### Analysis of results

Following Table 6, we highlight some strengths and weaknesses identified during the benchmark.

#### Technology-specific findings

*EMF tools are suitable for model validation* As expected, EMF tools perform well in model validation and transformation. EMF was designed to serve as a common basis for various MDE tools with in-memory model representation to improve performance. In principle, their in-memory nature may hinder scalability due to the memory limit of a single workstation, but despite this, some EMF solutions were among the most scalable ones.

**Table 6:** Summary of findings: strengths $\oplus$ and weaknesses $\ominus$

Domain of findings	Area	Observations
Technology	EMF	$\oplus$ EMF tools are suitable for model validation
		$\ominus$ No built-in indexing support in EMF
	Graph databases	$\oplus$ Good storage performance for graph databases
	RDF databases	$\ominus$ Underperforming RDF systems
		$\ominus$ Slow inferencing in RDF4J
	Relational databases	$\oplus$ Fast model load and good scalability from SQLite
		$\ominus$ MySQL slowdown for complex queries
Approach	Incremental	$\oplus$ Incremental tools prevail for continuous validation
		$\ominus$ The scalability of incremental tools is limited by memory constraints
	Search-based	$\oplus$ Search-based tools scale well for large models and simple queries
		$\ominus$ Search-based tools face problems for complex queries
	Indexing	$\oplus$ Substantial effect of indexing on performance
	Query language features	$\ominus$ Long path expressions are hard to evaluate
	Performance	$\oplus$ Huge differences in runtime across technologies
	Size of modifications	$\oplus$ Noticeable differences between the Inject and Repair scenarios

*No built-in indexing support in EMF* EMF does not offer built-in indexing support which would allow the system to quickly load the model, but may hinder efficient query evaluation. Indexing would significantly help local search approaches with adaptive model-specific search plans [24, 92, 93].

*Good storage performance for graph databases* We benchmark Neo4j with both its *core API* and the *Cypher query language*. Both show similar performance characteristics, with the core API approach at least half an order of magnitude faster. For importing large datasets, the CSV import provides good performance and scalability (unlike the GraphML import), but it requires the user to manually map the graph to a set of CSV files. However, query performance of the Cypher engine has not yet reached the efficiency of other local search-based query engines (e.g. Viatra). As a workaround, complex queries can be optimized by manually implementing the search algorithms (using the core API), which is aligned with the recommendation in [67, Chapter 6: Graph Database Internals]. This enables Neo4j to handle complex queries (unlike relational databases, for instance).

*Underperforming RDF systems* The in-memory SPARQL query engines (Jena, RDF4J) are in the slowest third of the tools, which is unexpected, considering their performance on benchmarks for different workloads (see Sect. 5.2). In our previous experiments, openly available disk-based SPARQL engines were even slower; hence, they were excluded from the benchmark.

*Fast model load and good scalability from SQLite* The SQLite implementation serves as a baseline for a comparison with more sophisticated tools. However, SQLite is surprisingly fast in several configurations. This may indicate that other technologies still have a lot of potential for performance enhancements.

*MySQL slowdown for complex queries* MySQL is not able to evaluate the more complex queries efficiently which prevents it from scaling for large models.

#### Approach-specific findings

*Incremental tools prevail for continuous validation* Incremental tools are very well suited for performing continuous model validation due to their low runtime and robustness wrt. query complexity. The approach introduces an overhead during the read phase but enables the systems to perform quick transformation–recheck cycles.

*The scalability of incremental tools is limited by memory constraints* Due to the memory overhead of incremental tools, they are unable to evaluate queries on the largest models used in the benchmark.

*Search-based tools scale well for large models and simple queries* Non-incremental tools are able to scale well by evaluating simple and moderately difficult queries even for the largest models of the benchmark. However, revalidation takes well over 1 s for large models of 1M+ elements.

*Search-based tools face problems for complex queries* For complex queries (ConnectedSegments and SemaphoreNeighbor), most non-incremental tools are unable to scale for large models (Fig. 8).

*Substantial effect of indexing on performance* As observed for some tools, such as Viatra Query (local search) and Neo4j, indexing has a substantial positive effect on performance. Using indexers allows Viatra Query to outperform the native EMF API solution, which lacks built-in indexing.

*Long path expressions are hard to evaluate* The ConnectedSegments query defines a long path expression: it looks for a sensor that has 6 segments (segment1, ..., segment6), connected by connectsTo edges. The results show that this query is quite difficult to evaluate Fig. 8. For RDF tools, queries using either *property paths* or metamodel-level *property chains* could lead to better performance. However, even though they are part of the SPARQL 1.1 [97] and the OWL 2 [57] standard, respectively, these features are not supported by most of the tools.

*Huge differences in runtime across technologies* While the overall characteristics of all tools are similar (low-order polynomial with a constant component), there is a rather large variation in execution times (with differences up to 4 orders of magnitude in revalidation time). This confirms our expectation that the persistence format, model load performance, query evaluation strategy and transformation techniques can have a significant impact on overall performance and deficiencies in any of these areas likely have a negative effect.

*Noticeable differences between the* Inject *and* Repair *scenarios.* As noted in Sect. 3.5, the main difference between the Inject and Repair scenarios is the number of model changes, which is significantly larger for the Repair scenario. The query result sets are also larger for the Repair scenario. By comparing corresponding plots, we observe that the overall evaluation time is affected linearly by this difference, meaning that all tools are capable of handling this efficiently.

### Threats to validity

#### Internal threats

*Mitigating measurement risks* To mitigate *internal validity threats*, we reduced the number of uncontrolled variables during the benchmark. Each measurement consisted of multiple runs to warm up the Java Virtual Machine and to mitigate the effect of transient faults such as noise caused by running our measurements in a public cloud environment.

*Ensuring functional equivalence and correctness* Queries are defined to be *semantically equivalent* across all query languages, i.e. for a particular query on a particular graph (defined by its scenario and size), the result set must be identical for all representations. To ensure the correctness of a solution, we specified tests for each query and transformation which were implemented and evaluated for all tools.

*Code reviews* To ensure comparable results, the query implementations were reviewed by experts of each technology as listed in “Acknowledgements” section.

*Search plans* The EMF API, the Neo4j Core API and the TinkerGraph implementations required a custom search plan. For each query, we used the same search plan in both implementations. As mentioned in Sect. 2.3.1, the search plans are not fully optimized, i.e. they are similar to what a developer would implement without fine-tuning performance. Our measurements exclude approaches with adaptive model-specific search plans [24, 92, 93], which were reported to visit fewer nodes (thus achieve lower execution time) compared to local search approaches with fixed search plans.

*In-memory versus disk-resident tools* As shown in Table 2, some of the tools use in-memory engines while others persist data on the disk. Even with SSD drives, memory operations are still more than an order of magnitude faster than disk operations, which favors the execution time of in-memory engines.

*Memory overhead introduced by the framework* To ensure deterministic results (see Sect. 3.8), the framework creates a copy of the match sets returned by the query engine. This introduces memory overhead by the framework itself. However, as the match sets are generally small compared to the size of the model (see Table 4), this overhead is negligible.

*Involvement in one of the tools* Several authors of the current paper are involved in the research and development of Viatra Query. However, since several queries originate from AUTOSAR validation constraints (see Sect. 3.4), this guarantees independence from the tools.

#### External threats

*Generalizability of results* Considering *external validity*, the most important factor is the relevance to real use cases. Based on our past experience in developing tools for critical systems [33], we believe that the metamodel (Sect. 2.2), the queries (Sect. 3.4) and the transformations (Sect. 3.5) are representative to models and languages used for designing critical embedded systems. Furthermore, as mentioned in Sect. 1, we believe the findings could be useful for other use cases with *similar workload profiles* that could benefit from incremental query evaluation.

The iterative revalidation with consecutive runs also follows the *commit-time source code analysis scenario* of [87].

### Summary

Finally, we revisit our research questions:

1. *How do existing query technologies scale for a continuous model validation scenario?*
  Most scalable techniques have low memory consumption in order to load large models. However, few query technologies are able to evaluate the queries and transformations required for model validation on graphs with more than 5 million elements.
2. *What technologies or approaches are efficient for continuous model validation?*
  Incremental query engines (like Viatra Query) are well suited to continuous validation workload by providing very low execution time, but their scalability is limited by increased memory consumption.
3. *What types of queries serve as performance bottleneck for different tools?*
  Queries with many navigations and negative constraints are a serious challenge for most existing tools.

## Related work

Numerous benchmarks have been proposed to measure and compare the performance of query and transformation engines in a specific technological space and a given use case. However, no openly available cross-technology benchmarks have been proposed for a continuous model validation scenario. Below we overview the main existing benchmarks for model query and transformation (Sect. 5.1) as well as RDF technologies (Sect. 5.2).

### Model transformation and graph transformation benchmarks

#### Graph transformation benchmarks

Up to our best knowledge, the first transformation benchmark was proposed in [91], which gave an overview on typical application scenarios of graph transformations together with their characteristic features. The paper presents two cases: the *Petri net firing simulation* case and the *object-relational mapping by model synchronization* case. While both are capable of evaluating certain aspects of incremental query performance, they provide a different workload profile (e.g. model and query characteristics) than typical well-formedness validation scenarios. [25] suggested some improvements to the benchmarks of [91] and reported measurement results for many graph transformation tools. Early benchmarks used much smaller models and more simple queries.

#### Tool contests

Many transformation challenges have been proposed as cases for graph and model transformation contests. Most of them do not focus on query performance; instead, they measure the usability of the tools, the conciseness and readability of the query languages and tests various advanced features, including reflection and traceability. The 2007 contest was organized as part of the AGTIVE conference [74], while the 2008 and 2009 contests were held during the GRaBaTS workshop [36, 65]. The contests in 2010, 2011, 2013, 2014 and later were organized as a separate event, the Transformation Tool Contest (TTC) [48, 69, 88, 89].

**Table 7:** Model transformation benchmarks. Notation for the *Performance-oriented* column: $\CIRCLE$ the case focuses on the performance of tools, $\LEFTcircle$ the case takes the performance into consideration but it is not the main focus, $\Circle$ the performance of the tools is mostly irrelevant for solving the case

Year	Case	Goal	Scope	t2m	m2m	m2t	Updates	Performance-oriented
2015	The Train Benchmark for Incremental Validation	Perform well-formedness validations and quick fix-like repair transformations.	Validation and modification	$\Circle$	$\CIRCLE$	$\Circle$	$\CIRCLE$	$\CIRCLE$
	The Model Execution Case	Define a transformation, which specifies the operational semantics of the UML activity diagram language by updating the runtime state of executed UML activity diagrams.	Model execution	$\Circle$	$\CIRCLE$	$\Circle$	$\Circle$	$\LEFTcircle$
	The Java Refactoring Case	Parse the source code, perform refactoring operations (pull up method, create superclass) on the program graph and generate the source code.	Refactoring	$\CIRCLE$	$\CIRCLE$	$\CIRCLE$	$\Circle$	$\Circle$
	Java Annotations (Live)	Use annotations to extend existing Java code.	Refactoring	$\CIRCLE$	$\CIRCLE$	$\CIRCLE$	$\Circle$	$\Circle$
2014	FIXML to Java, C# and C++	Transform financial transaction data expressed in FIXML format into class definitions in Java, C# and C++.	Deserialization	$\CIRCLE$	$\CIRCLE$	$\CIRCLE$	$\Circle$	$\Circle$
	Movie Database	Determine all actor couples who performed together in a set of at least three movies.	Model modification	$\Circle$	$\CIRCLE$	$\Circle$	$\Circle$	$\CIRCLE$
	The Transformation Tool Soccer Worldcup (Live)	Implement a soccer client, using model transformations.	AI	$\Circle$	$\CIRCLE$	$\Circle$	$\CIRCLE$	$\Circle$
2013	Petri-Nets to Statecharts	Mapping from Petri-Nets to statecharts.	Model synthesis	$\Circle$	$\CIRCLE$	$\Circle$	$\Circle$	$\CIRCLE$
	Class diagram restructuring	Perform refactoring operations: pull up, create superclass, create subclass.	Program refactoring	$\Circle$	$\CIRCLE$	$\Circle$	$\Circle$	$\Circle$
	Flowgraphs	Analysis and transformations in compiler construction: working on the data structures, control flow and data flow graphs.	Model synthesis and validation	$\Circle$	$\CIRCLE$	$\CIRCLE$	$\Circle$	$\Circle$
2011	GMF Model Migration	Migrate models in response to metamodel adaptation.	Model migration	$\Circle$	$\CIRCLE$	$\Circle$	$\Circle$	$\Circle$
	Compiler Optimization	Use the intermediate representation of the code to perform local optimizations, and do instruction selections to transform the intermediate representation to a least cost target representation.	Compiler optimization	$\Circle$	$\CIRCLE$	$\Circle$	$\Circle$	$\CIRCLE$
	Program Understanding: Reengineering	Create a state machine model out of a Java syntax graph.	Model synthesis	$\Circle$	$\CIRCLE$	$\Circle$	$\Circle$	$\LEFTcircle$
	Hello World	Several primitive tasks that can be solved straight away with most transformation tools.	Various	$\Circle$	$\CIRCLE$	$\CIRCLE$	$\CIRCLE$	$\Circle$
2010	Model Migration	Define a transformation to migrate the activity diagrams from UML 1.4 to UML 2.2.	model migration	$\Circle$	$\CIRCLE$	$\Circle$	$\Circle$	$\Circle$
	Topology Analysis of Dynamic Communication Systems	Compute the topologies that may occur for the merge protocol, a communication protocol which is used in car platooning.	Model synthesis	$\Circle$	$\CIRCLE$	$\Circle$	$\Circle$	$\CIRCLE$
	Ecore to GenModel	Use m2m transformation to synthesize the GenModel from the Ecore metamodel.	Model synthesis	$\Circle$	$\CIRCLE$	$\Circle$	$\Circle$	$\Circle$
2009	BPMN to BPEL Model Transformation	Define model transformations between BPMN and BPEL.	Model synthesis	$\Circle$	$\CIRCLE$	$\Circle$	$\Circle$	$\Circle$
	Program Comprehension	Perform (1) a simple filtering query on large models, (2) a complex query on small models, resulting in control flow graph and program dependence graph.	Model synthesis	$\Circle$	$\CIRCLE$	$\Circle$	$\Circle$	$\CIRCLE$
	Leader Election	Model and validate a simple leader election protocol using graph transformation rules, verification and testing.	Model verification	$\Circle$	$\CIRCLE$	$\Circle$	$\Circle$	$\Circle$
	Live Challenge Problem	Model a luggage system, define a transformation from a luggage system to a statechart model and perform simulation on the statechart model.	Model synthesis and simulation	$\Circle$	$\CIRCLE$	$\Circle$	$\Circle$	$\Circle$
2008	Program Refactoring	Import the models to GXL, allow for interactive transformations, export to GXL.	Program refactoring	$\Circle$	$\CIRCLE$	$\Circle$	$\Circle$	$\Circle$
	AntWorld	Perform a simulation of ants searching for food based on a few simple rules.	Model simulation	$\Circle$	$\CIRCLE$	$\Circle$	$\CIRCLE$	$\CIRCLE$
2007	Don’t Get Angry; Ludo Board Game	Model and play a board game using graph transformation rules.	Various	$\Circle$	$\CIRCLE$	$\Circle$	$\Circle$	$\CIRCLE$
	UML to CSP Transformation	Perform a transformation from UML activity diagrams to Communicating Sequential Processes.	Model synthesis	$\Circle$	$\CIRCLE$	$\Circle$	$\Circle$	$\Circle$
	Sierpinski Triangle	Construct a Sierpinski triangle.	Construction	$\Circle$	$\CIRCLE$	$\Circle$	$\CIRCLE$	$\CIRCLE$

Table 7 presents an overview of tool contest cases from 2007 to 2015. We shortly summarize their goal, scope and show whether solving them requires text-to-model (t2m), model-to-model (m2m) or model-to-text (m2t) transformations. We also denote whether the solution needs to perform updates on the model and whether the case explicitly measures the performance of the tools.

For the sake of conciseness, we only discuss cases that are potentially useful for measuring the performance of incremental model validation, meaning that they (1) are *performance-oriented*, e.g. they included large models, complex patterns or both, (2) measure the *incremental performance*, i.e. perform updates on the model and reevaluate the patterns.

The *AntWorld* case study [99] requires the solution to perform a simulation of ants searching for food based on a few simple rules. The environment, the ants and the food are modeled as a graph, while the rules of the simulation are implemented with model transformation rules. Although this case study provides a complex queries and performs update operations on a large model, its workload profile is similar to a model simulation instead of a model validation scenario.

The *Sierpinski Triangle Generation* [26] is another well-known transformation case, used in [44]. The Sierpinski triangles are stored as a model and are generated using model transformations. The triangles can be modeled with a very simple metamodel, and the characteristics of the instance models are very different from typical models used in MDE. While the required transformations are complex, the semantics of the transformation does not resemble any real-world applications.

The GRaBaTS 2009 *Program Comprehension* paper was used in [75] to benchmark the scalability of model persistence and query evaluation of NoSQL data stores.

Other performance-oriented benchmarks include the *Movie Database* [34], the *Petri-Nets to Statecharts* [90] and the *Program Comprehension* [42] cases, but none of these perform update and reevaluation sequences on the model.

#### Assessment of incremental model queries

In [8, 9], we aimed to design and evaluate model transformation benchmark cases corresponding to various usage patterns for the purpose of measuring the performance of incremental approaches on increasing model sizes. We assessed a hybrid model query approach (which combines local search and incremental evaluation) in [35] on the *AntWorld* case study.

Queries are common means to implement source code analysis, but it is traditionally a batch (and not continuous) validation scenario. Nevertheless, the performance of both local search-based and incremental model queries is assessed in [87] for detecting anti-patterns in source code transformed to EMF models.

As model validation is an important use case of incremental model queries, several model query and/or validation tools have been assessed in incremental constraint validation measurements [21, 63].

### RDF benchmarks

There are several well-defined performance benchmarks for assessing the performance of RDF technologies (overviewed in Table 8).

**Table 8:** RDF benchmarks

Benchmark	Year	Instance models	Model domain	Number of classes	Number of properties	Largest model	Number of queries	Multiple users	Workload profile	Measurement goal	Updates
LUBM	2004	Synthetic	University	43	32	6.9M	14	$\Circle$	Simple queries	Inferencing performance	$\Circle$
Barton	2007	Real	Library	11	28	50M	7	$\Circle$	Library search	Query response time	$\Circle$
SP^2^Bench	2009	Synthetic	DBLP	8	22	1B+	12	$\Circle$	Publication research	Query response time	$\Circle$
BSBM	2009	Synthetic	e-commerce	8	51	150B	12	$\CIRCLE$	Multi-user query set	Query throughput	$\LEFTcircle$
DBpedia	2011	Real	DBpedia	8	1200	300M	25	$\Circle$	Query set	Query throughput	$\Circle$
LDBC SNB	2015	Synthetic	Social network	19	27	1B+	14	$\Circle$	Query set	Navigational pattern matching	$\CIRCLE$
Train Benchmark	2016	Synthetic	Railway	9	13	23M+	6	$\Circle$	Model validation	Query and transformation response time	$\CIRCLE$

Notation for the *Largest model* column: “M” stands for million, “B” stands for billion. Notation for the *Updates* column: $\CIRCLE$ measuring the performance of updates is an important aspect of the benchmark, $\LEFTcircle$ the benchmark uses updates, but the performance of reevaluation after updates is not an important aspect, $\Circle$ the benchmark does not consider updates

One of the first ontology benchmarks are the *Lehigh University Benchmark (LUBM)* [30], and its improved version, the *UOBM Ontology Benchmark* [47]. These are tailored to measure reasoning capabilities of ontology reasoners. Another early benchmark used the *Barton dataset* [1] for benchmarking RDF stores. The benchmark simulates a user browsing through the RDF Barton online catalog. Originally, the queries were formulated in SQL, but they can be adapted to SPARQL as well. However, the model size is limited (50M elements) and there are no updates in the model.

*SP* ^2^ *Bench* [73] is a SPARQL benchmark that measures the execution time of various queries. The goal of this benchmark is to measure the query evaluation performance of different tools for a single set of SPARQL queries that contain most language elements. The artificially generated data are based on the real-world DBLP bibliography; this way instance models of different sizes reflect the structure and complexity of the original real-world dataset. However, other model element distributions or queries were not considered, and the complexity of queries was not analyzed.

The *Berlin SPARQL Benchmark (BSBM)* [11] measures SPARQL query evaluation throughput for an e-commerce case study modeled in RDF. The benchmark uses a single dataset, but recognizes several use cases with their own set of queries. The dataset scales in model size (10 million–150 billion), but does not vary in structure.

In the *SPLODGE* [27] benchmark, SPARQL queries are generated systematically, based on metrics for a predefined dataset. The method supports distributed SPARQL queries (via the SERVICE keyword); however, the implementation scales only up to three steps of navigation, due to the resource consumption of the generator. The paper does not discuss the complexity of the instance model, and only demonstrates the adequacy of the approach demonstrated with the RDF3X engine.

The *DBpedia SPARQL benchmark* [50] presents a general SPARQL benchmark procedure, applied to the DBpedia knowledge base. The benchmark is based on query-log mining, clustering and SPARQL feature analysis. In contrast to other benchmarks, it performs measurements on actually posed queries against existing RDF data.

The Linked Data Benchmark Council (LDBC) recently developed the Social Network Benchmark [20], a cross-technology benchmark, which provides an interactive workload and focuses on navigational pattern matching (i.e. dominantly local traversal operations, starting from a specific node).

### The Train Benchmark

The Train Benchmark is a *cross-technology* macrobenchmark that aims to measure the performance of continuous model validation with graph-based models and constraints captured as queries. Earlier versions of the benchmark have been continuously used for performance measurements since 2012 (mostly related to the Viatra Query framework) in various papers [37, 38, 40, 78, 86]. Compared to our previous publications, this paper has the following novel contributions:

- The benchmark features three distinct scenarios: Batch, Inject and Repair, each capturing a different aspect of real-world model validation scenarios. Previous publications only considered one or two scenarios.
- In this paper, we investigate the performance of query sets. Previously, we only executed the individual queries separately.
- Previous publications only used tools from one or two technologies. In the current paper, we assess 10 tools, taken from four substantially different technological spaces. This demonstrates that our benchmark is technology independent; thus, the results provide potentially useful feedback for different communities.

Compared to other benchmarks, the Train Benchmark has the following set of distinguishing features:

- The workload profile follows a *real-world model validation scenario* by updating the model with changes derived by simulated user edits or transformations.
- The benchmark measures the performance of both initial validation and (more importantly) incremental revalidation.
- This *cross-technology benchmark* can be adapted to different model representation formats and query technologies. This is demonstrated by 10 reference implementations over four different technological spaces (EMF, graph databases, RDF and SQL) presented as part of the current paper.

The benchmark is also part of the benchmark suite used by the MONDO EU FP7 project, along with other query/transformation benchmarks, such as the ITM Factory Benchmark,^5^ the ATL Zoo Benchmark^6^ and the OpenBIM Benchmark.^7^

## Conclusions and future work

### Conclusions

In this paper, we presented the *Train Benchmark*, a framework for the definition and execution of benchmark scenarios for modeling tools. The framework supports the construction of benchmark test sets that specify the metamodel, instance model generation, queries and transformations, result collection and processing, and metric evaluation logic that are intended to provide an end-to-end solution. As a main added value, this paper contains a comprehensive set of measurement results comparing 10 different tools from four technological domains (EMF, graph databases, RDF, SQL). These results allow for both intra-domain and cross-technology tool comparison and detailed execution time characteristics analysis.

*Criteria for domain-specific benchmarks* We revisit how our benchmark addresses the criteria of [29].

1. *Relevance* The Train Benchmark measures the runtime for the continuous revalidation of well-formedness constraints used in many industrial and academic design tools. It considers two separate practical scenarios: small model changes for manual user edits, and larger changes for automated refactorings.
2. *Portability* We presented the results for 10 implementations from four different technological domains in this paper. There are multiple other implementations available in the repository of the project.
3. *Scalability* The size of underlying models ranges from 5000 to 19 million model elements (triples), while there are 6 queries of various complexity (with negative and positive constraints).
4. *Simplicity* A simplified, EMF version of the Train Benchmark was used as part of the 2015 Transformation Tool Contest [80] where experts of four other tools managed to come up with an implementation, which indirectly shows the relative simplicity of our benchmark. An earlier version of the Train Benchmark was also used in [93] to assess the efficiency of various search plans.

*Software engineering aspects* From a software engineering perspective, the Train Benchmark has been continuously developed and maintained since 2012. The benchmark is available as an open-source project at https://github.com/FTSRG/trainbenchmark, implemented in Java 8. The project has end-to-end automation [38] to (1) set up configurations of benchmark runs, (2) generate large model instances, (3) execute benchmark measurements, (4) analyze the results and synthesize diagrams using R scripts [82]. The project provides continuous integration using the Gradle build system [28] and contains automated unit tests to check the correctness of the implementations.

### Future work

In the near future, we will add more implementations to the benchmark from all domains, including Epsilon [58] (EMF), OrientDB [55] (property graphs), PostgreSQL [49] (SQL) and INSTANS [66] (RDF).

Based on our previous work [40, 79], we plan to further investigate the connection between metrics (query metrics, model metrics and query on model metrics) and query performance.

## Specification of queries and transformations

### ConnectedSegments

*Description* The ConnectedSegments constraint requires that each sensor must have at most five segments attached to it. Therefore, the query (Fig. 7e) checks for sensors (sensor) that have at least six segments (segment1, ..., segment6) attached to them.

*Relational calculus formula*

$$\begin{aligned}&\big \{ \mathsf{sensor, segment1, segment2, segment3}, \\&\quad \mathsf{segment4, segment5, segment6} \big | \\&\quad \mathsf{Sensor(sensor)} \wedge \\&\quad \mathsf{Segment(segment1)} \wedge \mathsf{Segment(segment2)} \wedge \\&\quad \mathsf{Segment(segment3)} \wedge \mathsf{Segment(segment4)} \wedge \\&\quad \mathsf{Segment(segment5)} \wedge \mathsf{Segment(segment6)} \wedge \\&\quad \mathsf{connectsTo(segment1, segment2)} \wedge \\&\quad \mathsf{connectsTo(segment2, segment3)} \wedge \\&\quad \mathsf{connectsTo(segment3, segment4)} \wedge \\&\quad \mathsf{connectsTo(segment4, segment5)} \wedge \\&\quad \mathsf{connectsTo(segment5, segment6)} \wedge \\&\quad \mathsf{monitoredBy(segment1, sensor)} \wedge \\&\quad \mathsf{monitoredBy(segment2, sensor)} \wedge \\&\quad \mathsf{monitoredBy(segment3, sensor)} \wedge \\&\quad \mathsf{monitoredBy(segment4, sensor)} \wedge \\&\quad \mathsf{monitoredBy(segment5, sensor)} \wedge \\&\quad \mathsf{monitoredBy(segment6, sensor)} \big \} \end{aligned}$$

*Inject transformation.* A random segment segment1 is selected. The connectsTo edge running from segment1 to segment3 is deleted. A new segment segment2 is created and connected from segment1 to segment3. segment2 is also connected to the nodesensor, connected to segment1 via a sensor edge.

*Repair transformation.* The segment2 node and its edges are deleted from the matches. The segment1 and segment3 nodes are connected with a connectsTo edge.

### PosLength

*Description* The PosLength constraint requires that a segment must have a positive length. Therefore, the query (Fig. 7a) checks for segments (segment) with a length less than or equal to zero.

*Relational calculus formula*

$$\begin{aligned} \big \{ \mathsf{segment, length} \big | \mathsf{Segment(segment, length)} \wedge \mathsf{length} \le 0 \big \} \end{aligned}$$

*Inject transformation* The length property of randomly selected segments is updated to 0.

*Repair transformation* The length property of the segment in the match is updated to $-\mathsf {length}+1$.

### RouteSensor

The Inject and Repair transformations for the RouteSensor query are discussed in Sect. 3.6.

*Relational calculus formula*

$$\begin{aligned}&\big \{\mathsf{route, sensor, swP, sw} \big | \\&\quad \mathsf{Route(route)} \wedge \mathsf{Sensor(sensor)} \wedge \\&\quad \big (\exists \mathsf{currentPosition: SwitchPosition(swP, currentPosition) }\wedge \\&\quad \big (\exists \mathsf{position: Switch(sw, position)} \wedge \\&\quad \mathsf{follows(route, swP)} \wedge \mathsf{target(swP, sw)} \wedge \\&\quad \mathsf{monitoredBy(sw, sensor)} \wedge \lnot \mathsf{requires(route, sensor)} \big ) \big ) \big \} \end{aligned}$$

### SemaphoreNeighbor

*Description* The SemaphoreNeighbor constraint requires that the routes that are connected through sensors and track elements have to belong to the same semaphore. Therefore, the query (Fig. 7f) checks for routes (route1) which have an exit semaphore (semaphore) and a sensor (sensor1) connected to a track element (te1). This track element is connected to another track element (te2) which is connected to another sensor (sensor2) which (partially) defines another, different route (route2), while the semaphore is not on the entry of this route (route2).

*Relational calculus formula*

$$\begin{aligned}&\big \{ \mathsf{semaphore, route1, route2, sensor1, sensor2, te1, te2 }\big | \\&\quad \mathsf{Semaphore(semaphore)} \wedge \\&\quad \mathsf{Route(route1)} \wedge \mathsf{Route(route2)} \wedge \\&\quad \mathsf{Sensor(sensor1)} \wedge {Sensor(sensor2)} \wedge \\&\quad \mathsf{TrackElement(te1)} \wedge {TrackElement(te2)} \wedge \\&\quad \mathsf{exit(route1, semaphore)} \wedge {requires(route1, sensor1)} \wedge \\&\quad \mathsf{monitoredBy(te1, sensor1)} \wedge {connectsTo(te1, te2)} \wedge \\&\quad \mathsf{monitoredBy(te2, sensor2)} \wedge {requires(route2, sensor2)} \wedge \\&\quad \lnot \mathsf{entry(route2, semaphore)} \big \} \end{aligned}$$

*Inject transformation* Errors are introduced by disconnecting the entry edge of the selected routes. (According to the metamodel, a route may only have 0 or 1 entry edges.)

*Repair transformation* The route2 node is connected to the semaphore node with an entry edge.

### SwitchMonitored

*Description* The SwitchMonitored constraint requires that every switch must have at least one sensor connected to it. Therefore, the query (Fig. 7b) checks for switches (switch) that have no sensors (sensor) associated with them.

*Relational calculus formula*

$$\begin{aligned}&\big \{ \mathsf{sw} \big | \mathsf{Switch(sw)} \wedge \\&\quad \left( \lnot \exists \mathsf{sensor: Sensor(sensor)} \wedge \mathsf{monitoredBy(switch, sensor)}\right) \big \} \end{aligned}$$

*Inject transformation* Errors are injected by randomly selecting switches (switch) and deleting all their edges to sensors. If the selected switch was invalid, it did not have such an edge, so no edges are deleted and the switch stays invalid. If the chosen switch was valid, it will become invalid.

*Repair transformation* A sensor is created and connected to the switch.

### SwitchSet

*Description* The SwitchSet constraint requires that an entry semaphore of a route may only show GO if all switches along the route are in the position prescribed by the route. Therefore, the query (Fig. 7d) checks for routes (route) which have an entry semaphore (semaphore) that shows the GO signal. Additionally, the route follows a switch position (swP) that is connected to a switch (sw), but the switch position (swP.position) defines a different position from the current position of the switch (sw.currentPosition).

*Relational calculus formula*

$$\begin{aligned}&\big \{ \mathsf{semaphore, route, swP, sw, currentPosition, position} \big | \\&\quad \mathsf{Route(route)} \wedge \mathsf{SwitchPosition(swP, position)} \wedge \\&\quad \mathsf{Switch(sw, currentPosition)} \wedge \mathsf{currentPosition} \ne \mathsf{position} \wedge \\&\quad \mathsf{Semaphore(semaphore, ``GO'')} \wedge \mathsf{entry(route, semaphore)} \wedge \\&\quad \mathsf{follows(route, swP)} \wedge \mathsf{target(swP, sw)} \big \} \end{aligned}$$

*Inject transformation* Errors are injected by randomly selecting switches (switch) and setting their currentPosition property to the next enum value, e.g. from LEFT to RIGHT.

*Repair transformation* The currentPosition property of switch is set to the position of swP.

## Footnotes

1
*Microbenchmarks* measure the performance of primitive operations supported by an underlying platform, often focusing on the performance of a single method. *Macrobenchmarks* test complex use cases derived from real applications [13, 98].

2 The implementation and the detailed results are available online at http://docs.inf.mit.bme.hu/trainbenchmark.

3
http://docs.inf.mit.bme.hu/trainbenchmark.

4 We excluded MySQL from this measurement as limiting its available memory only causes it to use the disk more extensively, so this method cannot give a good approximation on its memory consumption. We also excluded SQLite as it uses the native heap instead of the Java heap.

5
https://github.com/atlanmod/mondo-itmfactory-benchmark.

6
https://github.com/atlanmod/mondo-atlzoo-benchmark.

7
https://github.com/atlanmod/mondo-openbim-benchmark.
